# Systemic microvascular dysfunction in microvascular and vasospastic angina

**DOI:** 10.1093/eurheartj/ehy529

**Published:** 2018-08-27

**Authors:** Thomas J Ford, Paul Rocchiccioli, Richard Good, Margaret McEntegart, Hany Eteiba, Stuart Watkins, Aadil Shaukat, Mitchell Lindsay, Keith Robertson, Stuart Hood, Eric Yii, Novalia Sidik, Adam Harvey, Augusto C Montezano, Elisabeth Beattie, Laura Haddow, Keith G Oldroyd, Rhian M Touyz, Colin Berry

**Affiliations:** 1 West of Scotland Heart and Lung Centre, Golden Jubilee National Hospital, GJNH, Agamemnon St, Glasgow, UK; 2 British Heart Foundation Glasgow Cardiovascular Research Centre, Institute of Cardiovascular and Medical Sciences, University of Glasgow, 126 University Place, University of Glasgow, Glasgow, UK; 3 Faculty of Medicine, University of New South Wales, Sydney, NSW, Australia

**Keywords:** Endothelial function, Angina pectoris, Coronary blood flow, Coronary microcirculation, Ischaemia, Vasospasm, Endothelin

## Abstract

**Aims:**

Coronary microvascular dysfunction and/or vasospasm are potential causes of ischaemia in patients with no obstructive coronary artery disease (INOCA). We tested the hypothesis that these patients also have functional abnormalities in peripheral small arteries.

**Methods and results:**

Patients were prospectively enrolled and categorised as having microvascular angina (MVA), vasospastic angina (VSA) or normal control based on invasive coronary artery function tests incorporating probes of endothelial and endothelial-independent function (acetylcholine and adenosine). Gluteal biopsies of subcutaneous fat were performed in 81 subjects (62 years, 69% female, 59 MVA, 11 VSA, and 11 controls). Resistance arteries were dissected enabling study using wire myography. Maximum relaxation to ACh (endothelial function) was reduced in MVA vs. controls [median 77.6 vs. 98.7%; 95% confidence interval (CI) of difference 2.3–38%; *P* = 0.0047]. Endothelium-independent relaxation [sodium nitroprusside (SNP)] was similar between all groups. The maximum contractile response to endothelin-1 (ET-1) was greater in MVA (median 121%) vs. controls (100%; 95% CI of median difference 4.7–45%, *P* = 0.015). Response to the thromboxane agonist, U46619, was also greater in MVA (143%) vs. controls (109%; 95% CI of difference 13–57%, *P* = 0.003). Patients with VSA had similar abnormal patterns of peripheral vascular reactivity including reduced maximum relaxation to ACh (median 79.0% vs. 98.7%; *P* = 0.03) and increased response to constrictor agonists including ET-1 (median 125% vs. 100%; *P* = 0.02). In all groups, resistance arteries were ≈50-fold more sensitive to the constrictor effects of ET-1 compared with U46619.

**Conclusions:**

Systemic microvascular abnormalities are common in patients with MVA and VSA. These mechanisms may involve ET-1 and were characterized by endothelial dysfunction and enhanced vasoconstriction.

**Clinical trial registration:**

ClinicalTrials.gov registration is NCT03193294.

**Figure:**
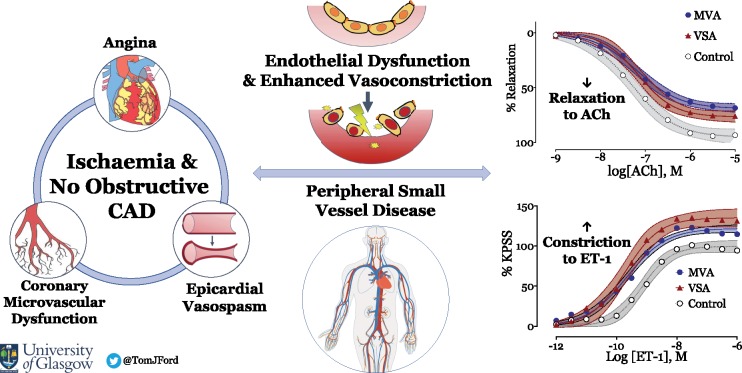

## Introduction

**Figure 1:**
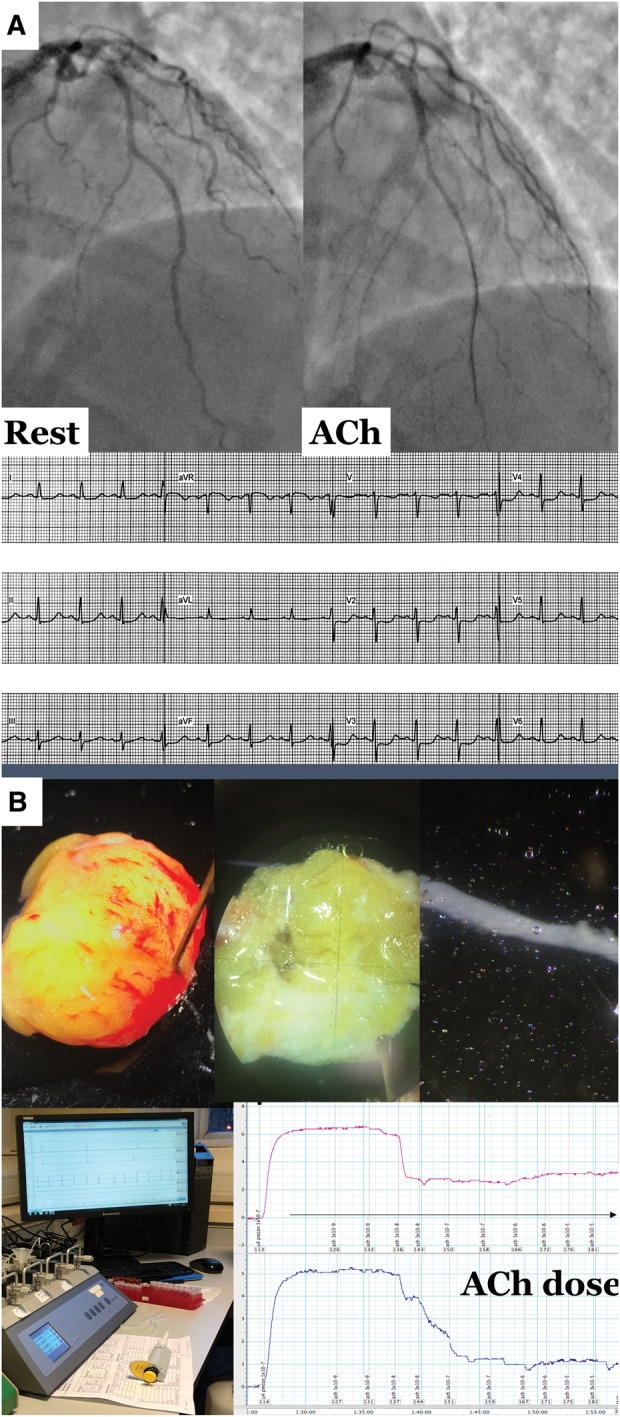
(*A*) Illustrative case of microvascular angina. (*B*) Gluteal biopsy procedure, dissection of resistance artery, and myography workstation. (*A*) A 43-year-old female smoker with family history of early cardiovascular disease was referred for invasive angiography with 12 months of typical angina and positive stress ECG. Recruited into British Heart Foundation CorMicA study with findings of non-obstructive atheroma in the left anterior descending coronary artery. Pressure wire assessment confirmed profoundly reduced coronary flow reserve (1.3) but non-obstructive physiology (fractional flow reserve 0.86) and normal index of microvascular resistance with adenosine (index of microcirculatory resistance 18). Acetylcholine testing confirmed microvascular spasm to acetylcholine (reproduction of typical angina, ST segment deviation and <90% epicardial vasoconstriction to acetylcholine. (*B*) Surgical gluteal skin fat biopsy with dissection of resistance artery under light microscopy. Peripheral resistance arteries were harvested and set-up for our wire myography protocol. The myography tracing shows a cumulative concentration-response curve to dilator agonist acetylcholine. We averaged the relaxation at each concentration compared to baseline confirming reduced maximal relaxation to this probe of endothelial function. Overall this lady was diagnosed with microvascular angina (typical angina, proven coronary microvascular dysfunction) with evidence of widespread peripheral endothelial dysfunction.

Coronary microvascular dysfunction and/or vasospasm are potential causes of ischaemia in patients with no obstructive coronary artery disease (INOCA).^1^ Patients with INOCA have unmet clinical needs with high morbidity, impaired quality of life, and health resource utilization.^2^^,^^3^ Relevant tests to determine the presence of coronary microvascular dysfunction are rarely performed during invasive coronary angiography, but they may provide the diagnosis (*Figure 1A*). The 2013 European Society of Cardiology (ESC) guidelines on the management of stable coronary artery disease (CAD) were notable as the first international practice guidelines to address the diagnosis and management of functional coronary disorders.^4^

Importantly, microvascular disease may be a multi-system entity with links between coronary microvascular dysfunction and small vessel disease of the kidney,^5^ retina,^6^ and cerebral white matter.^7^ Indeed, systemic endothelial impairment has been suggested as a key mediator in the pathogenesis of coronary microvascular dysfunction.^8^^,^^9^ The technique of wire myography has been used to investigate peripheral vascular dysfunction in conditions such as hypertension,^10^ heart failure,^11^ and renal failure^12^ (*Figure 1B*). We hypothesised that small vessel damage in the heart may be a systemic phenomenon and that patients have generalized endothelial dysfunction leading to abnormal vascular reactivity assessed using wire myography. To test this hypothesis, we designed a case–control study to investigate peripheral small artery changes in two distinct groups of INOCA—those with microvascular angina (MVA) and those with vasospastic angina (VSA). Our aim was to compare peripheral endothelial function and vascular reactivity in these two groups with matched control subjects (chest pain but normal invasive assessment of coronary microvascular function).

## Methods

### Study population

We screened elective adult referrals to two large cardiac catheterization laboratories with clinical suspicion of significant coronary artery disease based on the presence of angina. Patients were prospectively recruited from the larger British Heart Foundation Coronary Microvascular Angina (CorMicA) study which is a randomized controlled trial evaluating the effect of diagnostic strategy and linked therapy on symptoms and quality of life in INOCA.^13^ The Rose–Angina questionnaire was administered on the day of the angiogram and only patients with definite or possible angina were eligible for the study.^14^ Exclusion criteria included a non-coronary indication for invasive angiography e.g. valve disease, severe renal dysfunction (GFR <30 mL/min), inability to give informed consent, and obstructive coronary disease on coronary angiography [≥50% diameter stenosis and/or fractional flow reserve (FFR) ≤0.80]. All coronary vasodilating drugs were discontinued at least 24 h before coronary artery function testing.

### Definitions: microvascular angina, vasospastic angina, and control subject

We defined MVA according to the COVADIS diagnostic criteria: stable angina,^14^ unobstructed coronary arteries and proven coronary microvascular dysfunction (*Table 1*). The CorMicA study design paper describes the coronary artery function testing procedure.^13^ A total of 11 control subjects were recruited: 9 from the CorMicA study (NCT03193294) and 2 from a contemporaneous related study (CorCTCA: NCT03477890). Control subjects had stable chest pain syndromes investigated with the same diagnostic protocols but coronary microvascular dysfunction was not found during invasive interrogation. Coronary microvascular dysfunction during acetylcholine (ACh) (microvascular spasm) was defined as reproduction of angina, ischaemic electrocardiogram (ECG) shifts, but no epicardial spasm during ACh testing.^27^ Patients without coronary microvascular dysfunction but with significant epicardial vasoconstriction (≥90%) during ACh testing along with (i) reproduction of the usual chest pain and (ii) ischaemic ECG changes, were diagnosed with VSA according to the COVADIS criteria and formed a comparator group.^15^

**Table 1:** Definitions of abnormalities in coronary artery function

	Disorder of coronary artery function	
CMD	↑ Microvascular resistance (IMR ≥25)	The IMR represents a quantitative metric of microvascular function independent of resting haemodynamics
		IMR = distal coronary pressure * transit time (average time for 3 saline bolus runs at hyperaemia)
	↓ Coronary vasorelaxation (CFR <2.0)	CFR by thermodilution. A CFR<2.0 reflects the failure to increase coronary flow above two times the resting flow in response to a hyperaemic stimulus
	Microvascular spasm	Reproduction of usual angina during intracoronary infusion of ACh with ischaemic ST-segment changes but no significant epicardial coronary constriction (<90% reduction in epicardial coronary diameter). Represents inappropriate susceptibility to microvascular constriction
VSA	Epicardial spasm	Epicardial coronary artery spasm is defined as ≥90% reduction in coronary diameter following intracoronary administration of ACh (100 µg) Reproduction of usual symptoms ST segment deviation on the ECG
Non-cardiac (control)	Nil	Exclusion of diffuse or obstructive epicardial coronary disease (FFR >0.8)
		Normal invasive metrics of coronary artery function: CFR ≥2.0 IMR <25 Negative provocation testing (ACh)

ACh, acetylcholine; CFR, coronary flow reserve; CMD, coronary microvascular dysfunction; FFR, fractional flow reserve; IMR, index of microcirculatory resistance; INOCA, ischaemia and non-obstructive coronary artery disease; MVA, microvascular angina; VSA, vasospastic angina.

### Coronary artery function testing

In brief, we measured coronary flow reserve (CFR) and the index of microcirculatory resistance (IMR) using thermodilution as previously described.^16^^,^^17^ An intravenous infusion of adenosine (140 μg·kg^−1^·min^−1^) was administered via a large peripheral vein to induce steady-state maximal hyperaemia. Increased IMR (≥25) was representative of microvascular dysfunction.^18^ Coronary flow reserve was calculated using thermodilution as resting mean transit time divided by hyperaemic mean transit time^19^ (abnormal CFR is defined as <2.0).^20^ Fractional flow reserve was calculated by the ratio of mean distal coronary pressure to mean aortic pressure at maximal hyperaemia—abnormal FFR is defined as ≤0.80.

### Coronary vasoreactivity testing

The target vessel was the left anterior descending coronary artery. If technical factors, e.g. vessel tortuosity, precluded assessment of this artery then the left circumflex or right coronary artery was selected. We assessed endothelium-dependent coronary vasomotor function using intra-coronary infusions of ACh via the guiding catheter at concentrations of 0.182, 1.82, and 18.2 µg/mL (10^−6^, 10^−5^, and 10^−4 ^mol/L, respectively) at 1 mL/min for 2 min via a mechanical infusion pump.^21^ We then performed provocation testing for epicardial coronary artery spasm using a 100 μg bolus of ACh (5.5 mL of 10^−4 ^mol/L over 20 s—reduced to 50 μg for the right coronary artery).

### Preparation of small resistance arteries

Vessels were dissected from a gluteal skin fat biopsy (approximately 3 × 2 × 2 cm) within 4 weeks of coronary angiography. Vasoactive medications were withheld for at least 24 hours prior to the surgical biopsy. Small resistance arterioles (normalized diameter <400 μm) were studied in a Mulvany-Halpern 4-channel wire myograph (Danish Myotech, Aarhus, Denmark) with isometric tension recordings made as previously described.^22^

### Experimental Protocol

After a standard normalization and start-up protocol involving repeated washes with high potassium chloride solution (62.5 mM), the arterioles were pre-constricted with thromboxane-A2 analogue (U46619; 0.1μM). Previous work on human resistance arteries support its application in myography due to its consistent vasoconstriction with a steady plateau from which to assess arteriolar relaxation. Blood vessels with no responses were discarded. We averaged the relaxation response to each incremental concentration of acetycholine (ACh; 1nM-3μM) in multiple arterioles in each subject. This served to increase accuracy by levelling out the regional heterogeneity of endothelial function in vivo.^23^^,^^24^ We then performed separate parallel experiments with cumulative concentration response curves (CCRCs) to the vasoconstrictors endothelin-1 (ET-1; 1pM-1 μM) and thromboxane-A2 analogue (U46619; 0.1nM-3 μM). After performing the U46619 CCRC, a vessel washout was performed and further preconstruction to U46619 allowed us to examine the effects of endothelial independent vasodilator, sodium nitroprusside (SNP; 0.1nM-3μM). Due to the irreversible nature of ET-1 receptor binding, arterioles subjected to ET-1 CCRC were not used for further experiments. Cumulative concentrationresponse curves were fitted using four-parameter, non-linear regression curve fitting in Prism 7.0 (GraphPad Inc, La Jolla, CA, USA). Maximum efficacy (E_max_) for vasoconstrictors was expressed as a percentage of the mean response of the contraction to 62.5mM potassium chloride solution. For relaxation data, the maximum response (E_max_) to ACh was expressed as percentage relaxation after preconstruction with thromboxane analogue (U46619; 0.1μM). Sensitivity of the arterioles to each compound was expressed as the pEC50 (constrictors) or pIC50 (inhibitors) derived from the CCRC. Prism 7.0 The pEC50 value represents the minus log concentration required to produce 50% of the maximum response. Similarly, pIC50 is the -log of concentration required to inhibit 50% of the maximum response. Higher numbers indicate more potency (less concentration required to achieve the median response).

### Statistical analysis

The study population analysed comprised all of the participants who underwent a biopsy and no patients were excluded even if no viable vessels were dissected. The primary endpoint of this study was the difference in maximum relaxation induced by ACh (ACh *E*_max_) between two groups: MVA and control subjects. Sample size calculations were based on this measure. Using an allocation ratio of 5:1 (MVA:controls), we calculated that a sample size of 52 patients and 10 controls would have 80% power to detect between group difference in ACh *E*_max_ of 10% at the 5% significance level. A minimum sample size of 62 subjects was estimated using G*Power 3.1 (University of Melbourne, Parkville, Victoria, Australia). This calculation used the Mann–Whitney *U* test reflecting small sample size and likelihood of non-parametric distribution.^12^ A secondary analysis was performed to evaluate differences in potency and efficacy of dilator and constrictor agonists between all three groups. The Kruskal–Wallis test was used to evaluate this with an adjustment for multiple comparisons (controlling the false discovery rate).

Results are reported as mean (± standard deviation) for parametric data and median (25th and 75th percentile) for data that were not normally distributed. The Fisher’s exact test or the χ^2^ test was used to assess for differences between categorical variables. One-way analysis of variance was used to assess for differences between means of continuous normally distributed variables across three groups (adjusted for multiple comparisons). We applied the Shapiro–Wilk normality test to establish whether the data followed a parametric distribution. Best-fit cumulative concentration curves (CCRCs) were compared with the extra sum-of-squares *F* test. We performed two-tailed analysis and considered a *P*-value <0.05 to be significant. Statistical analyses were performed with Prism 7.0 (GraphPad, La Jolla, CA, USA) and SPSS 24.0 (SPSS, Chicago, IL, USA).

## Results

From December 2017—June 2018, 81 subjects gave informed consent and underwent clinically-indicated invasive coronary angiography coupled with guidewire-based measurements of coronary artery function and vasoreactivity testing. A gluteal skin fat biopsy was then obtained within 4 weeks of the angiogram. The mean age of the MVA patients was 63 years which was similar to the VSA and control subjects. Overall, the characteristics of the patients including demographics, risk factors, and treatment were similar between the groups (*Table 2*). Non-obstructive epicardial CAD severity, assessed using Gensini score^25^ and FFR, did not differ between the groups (*P* = 0.15). All 59 MVA patients had demonstrable evidence of coronary microvascular dysfunction including 50 (85%) with abnormal CFR and/or IMR, 23 (39%) with evidence of microvascular spasm to ACh and 14 (24%) with abnormalities to both adenosine and ACh. Coronary flow reserve was reduced in MVA (2.4 ± 1.1) vs. VSA (3.6 ± 2.2) and control subjects (3.7 ± 1.1; *P* = 0.001). Coronary microvascular resistance was greater in MVA (mean IMR 28.2 ± 16.3) compared with VSA (15.8 ± 1.6) and control subjects controls (16.6 ± 6; *P* = 0.005).

**Table 2:** Baseline demographics and invasive coronary artery function

	MVA (*n* = 59)	VSA (*n* = 11)	Control (*n* = 11)	*P*-value
Age (years)	63 (±10.2)	57 (±11)	61 (±8)	0.19
Female gender	40 (68)	8 (73)	8 (73)	0.91
Diabetes	9 (15)	1 (9)	1 (9)	0.77
Hypertension	42 (71)	6 (55)	7 (64)	0.74
Previous MI	12 (20)	0 (0)	0 (0)	0.07
Current smoker	10 (17)	1 (9)	1 (9)	0.68
BMI (kg/m^2^)	30 (±6.8)	25.6 (±8.9)	32.4 (±7.5)	0.08
Pulse (min)	71 (±13)	71 (±14)	69 (±13)	0.88
Systolic blood pressure (mmHg)	138 (±22)	133 (±25)	138 (±17)	0.72
Diastolic blood pressure (mmHg)	72 (±12)	73 (±16)	74 (±12)	0.84
Medications				
ACE-I	24 (41)	5 (45)	4 (36)	0.91
Beta blocker	40 (68)	7 (64)	6 (55)	0.69
CCB	20 (34)	5 (45)	4 (36)	0.76
Statin	49 (83)	10 (91)	7 (64)	0.22
Cholesterol (mmol/L)	3.5 (±1.0)	3.3 (±1.1)	4.2 (±1.7)	0.13
Glucose (mmol/L)	4.9 (±1.8)	4.3 (±1.2)	4.5 (±0.9)	0.42
hsCRP (mg/L)^a^	3.5 (±5.2)	1.3 (±1.0)	4.9 (±10.4)	0.39
Coronary angiography and invasive coronary physiology				
Angiographically normal	28 (47)	8 (73)	7 (64)	0.23
Gensini score	3.2 (±2.2)	1.6 (±2.2)	1.4 (±2.1)	0.15
LVEDP (mmHg)	10 (±4)	8 (±2)	8 (±2)	0.44
FFR	0.88 (±0.05)	0.88 (±0.05)	0.89 (±0.04)	0.77
Coronary microvascular dysfunction	59 (100)	0 (0)	0 (0)	<0.001
CFR and/or IMR	50 (85)	0 (0)	0 (0)	<0.001
IMR ≥25	31 (53)	0 (0)	0 (0)	<0.001
CFR <2.0	32 (55)	0 (0)	0 (0)	<0.001
CFR <2.5	41 (71)	3 (27)	1 (9)	<0.001
CMD to ACh	23 (39)	0 (0)	0 (0)	0.003
Both ACh and CFR/IMR	14 (24)	0 (0)	0 (0)	0.043
CFR	2.4 (±1.1)	3.6 (±2.2)	3.7 (±1.1)	0.001
IMR	28.2 (±16.3)	15.8 (±1.6)	16.6 (±6)	0.005

Data are represented as mean (±SD) and number (%). Includes all patients in intention-to-treat analysis. Significance determined as comparison between three groups by one-way analysis of variance or Pearson χ^2^ test for categorical variables adjusted using the Bonferroni correction for multiple comparisons.

ACE-I, angiotensin converting enzyme inhibitor; ACh, acetylcholine; BMI, body mass index; CCB, calcium channel blocker; CFR, coronary flow reserve; CMD, coronary microvascular dysfunction; CRP, C-reactive protein; FFR, fractional flow reserve; hsCRP, high sensitivity C-reactive protein; IMR, index of microcirculatory resistance; LVEDP, left ventricular end-diastolic pressure; MI, myocardial infarction.

a hsCRP results available in only 8 controls, 10 VSA, and 47 MVA.

In total, 81 biopsies were performed (59 MVA, 11 VSA and 11 controls). Three surgical biopsies had no functional small resistance arteries (all MVA patients). The normalized internal diameters of resistance arteries did not differ between MVA, VSA, and control subjects (mean diameter 345 vs. 332 vs. 315 µm, *P* = 0.43; *Table 3*). Additional information on vessels including length and number of viable vessels is demonstrated in *Table 4*.

**Table 3:** Maximum responses and sensitivities to dilator and constrictor agonists in resistance arteries from patients with microvascular angina, vasospastic angina, and control subjects with normal coronary function

	MVA (*n* = 59)		VSA (*n* = 11)		Control (*n* = 11)
		*P*-value^a^		*P*-value^a^	
Normalized vessel diameter (µm)^b^	345 (±95)	0.34	332 (±85)	0.66	315 (±96)
ACh					
*N*	48		9		10
*E*_max (%)_	77.6	<0.01	79.0	0.03	98.7
*pIC*_50_	7.1	0.49	7.1	0.73	7.3
SNP					
* N*	49		10		10
* E*_max (%)_	97	0.99	99	0.99	98
*pIC*_50_	7.0	0.50	6.5	0.30	7.5
ET-1					
*N*	54		11		9
* E*_max (%)_	121	0.03	125	0.02	100
*pEC*_50_	9.6	0.17	9.5	0.49	9.3
U44619					
*N*	54		10		11
* E*_max (%)_	143	0.01	141	0.04	109
*pEC*_50_	8.0	0.02	7.5	0.67	7.50

Data for diameter are represented as mean (±SD). Potency is expressed as the −log concentration required to produce 50% of the maximum response (IC_50_ for antagonists, EC_50_ for agonists).

ACh, acetylcholine; *E*_max_, maximum efficacy for constrictor agonists is expressed in terms of percentage of maximum response to KPSS solution, for dilator agonists *E*_max_ refers to maximum relaxation after preconstruction with U46619; SNP, sodium nitroprusside.

a Efficacy and potency were not significantly different between the MVA and VSA groups for any of the agonists studied. *P*-value represents two-tailed comparison of median from each group with median of the control group using Kruskal-Wallis test (adjusting for multiple comparisons using the false discovery rate)

b L_0_ = 90% of the normalized vessel diameter. *P*-value represents an unpaired t-test comparing vessel diameter of MVA versus control and VSA versus control.

**Table 4:** Vessel information

	MVA (*n* = 59)	VSA (*n* = 11)	Controls (*n* = 11)	*P*-value
*N* with viable vessels	56 (95%)	11 (100%)	11 (100%)	0.56
Vessels per subject	6.2 (1.9)	6.2 (±2.4)	5.7 (±2.3)	0.81
Length (mm)	1.85 (±0.1)	1.86 (±0.1)	1.82 (±0.1)	0.66
Acetylcholine				
Patients	48 (84%)	9 (82%)	10 (91%)	0.33
Vessels	176	32	22	
SNP				
Patients	49 (97%)	10 (91%)	10 (91%)	0.32
Vessels	58	16	15	
U46619				
Patients	54 (93%)	10 (91%)	11 (100%)	0.84
Vessels	75	16	15	
Endothelin-1				
Patients	54 (94%)	11 (100%)	9 (82%)	0.14
Vessels	54	12	10	

Patients = number of patients in whom a complete concentration-response curve was obtained for the agonist. Vessels refer to the number of vessels used in the group to average the response. Data are mean (±SD) with comparison by one-way analysis of variance.

### Responses to dilator agonists

**Figure 2:**
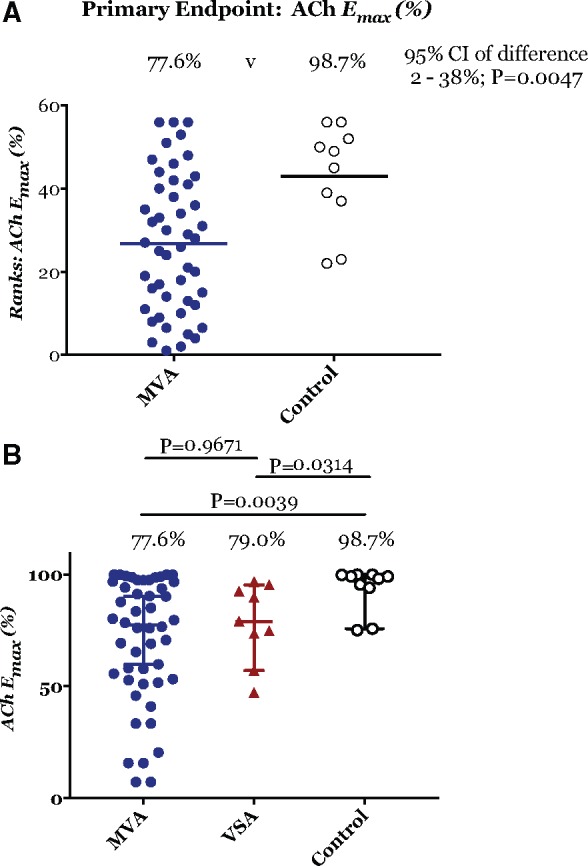
(*A*) Primary endpoint: maximum vasorelaxation to acetylcholine (*E*_max_). (*B*) Comparison of maximum relaxation to acetylcholine between the three groups. (*A*) Ranks: the Mann–Whitney *U* test ranking of microvascular angina vs. control subjects confirming significantly lower maximum vasorelaxation in response to acetylcholine between microvascular angina patients and control subject (median 77.6 vs. 98.7%; 95% confidence interval of difference between medians 2.3–38%; *P* = 0.0047). (*B*) Comparison of three groups confirming both microvascular angina subjects (blue circle; acetylcholine *E*_max_ 77.6%, *n* = 48) and vasospastic angina subjects (red triangle; acetylcholine *E*_max_ 79%, *n* = 9) had reduced maximum vasorelaxation to acetylcholine than vs. control subjects (○—white circle, *E*_max_ 98.7%, *n* = 10). Significance *P* < 0.01 and *P* = 0.03, respectively using the Kruskal–Wallis test (adjusted for multiple comparisons by controlling the false discovery rate). Each measure represents mean ± 95% confidence intervals for mean in shaded contours from CCRC best-fit. There were no significant differences in maximum relaxation to acetylcholine between microvascular angina and vasospastic angina subjects (*P* = 0.96).

**Figure 3:**
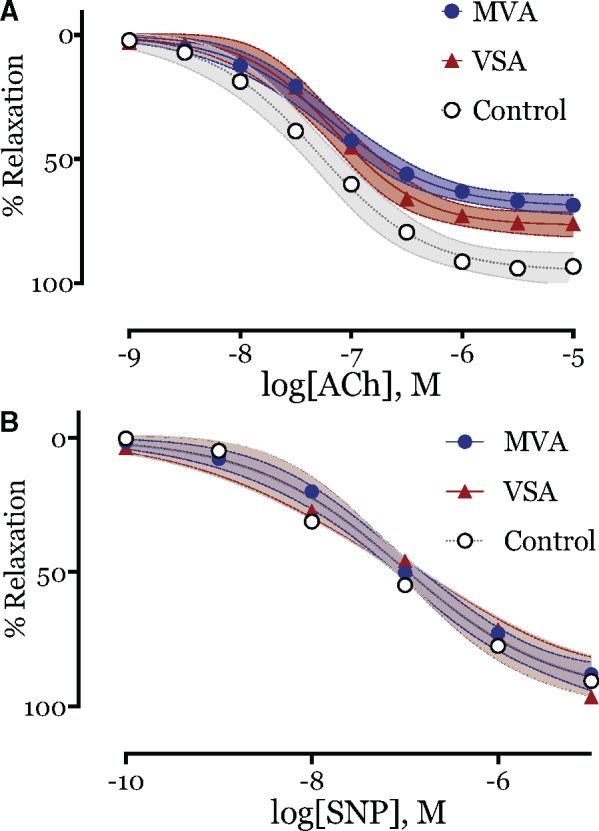
Cumulative concentration-response curves to dilator agonists. (*A*) Endothelial function assessed by acetylcholine showing impairment in vasorelaxation in both microvascular angina subjects (blue circle; acetylcholine *E*_max_ 77.6%, *n* = 48; *P* < 0.01) and vasospastic angina subjects (red triangle; acetylcholine *E*_max_ 79%, *n* = 9; *P* = 0.03) vs. control subjects (○—white circle, *E*_max_ 98.7%, *n* = 10). No difference in acetylcholine *E*_max_ between microvascular angina and vasospastic angina subjects (*P* = 0.967). Comparison of CCRC fit, *P* < 0.001. (*B*) No difference in vasorelaxation to endothelial independent probe, sodium nitroprusside between microvascular angina patients (blue circle, sodium nitroprusside *E*_max_ 97%, *n* = 49), vasospastic angina subjects (red triangle; acetylcholine *E*_max_ 99%; *n* = 10) and control subjects (○—white circle, *E*_max_ 98%, *n* = 10; *P* = 0.99). Each measure represents mean ± 95% confidence intervals for mean in shaded contours from CCRC best-fit. No difference in response to sodium nitroprusside between the groups, *P* = 0.4914.

Acetylcholine and SNP evoked concentration-dependent relaxations in preconstructed resistance arteries from all groups. For the primary study endpoint, the maximum relaxation to ACh was significantly lower in patients with MVA compared with controls [median 77.6% vs. 98.7%; 95% confidence interval (CI) of difference in medians 2–38%, Mann-Whitney U = 106, *P* = 0.0047; *Table 3* and *Figure 2A*]. The maximum relaxation to ACh was also lower in patients with VSA compared with controls (median 79.0 vs. 98.7%; *P* = 0.031; *Table 3* and *Figure 2B*). The maximum relaxation to ACh did not differ between patients with MVA and VSA (*P* = 0.967, *Figures 2B* and *3A*). Maximum relaxation to the endothelium-independent dilator, SNP, was similar in small resistance arteries from MVA patients compared with controls (97 vs. 98%; 95% CI of difference in medians −3.3 to 4.5%, *P* = 0.962; *Table 3* and *Figure 3B*).

### Responses to constrictor agonists

**Figure 4:**
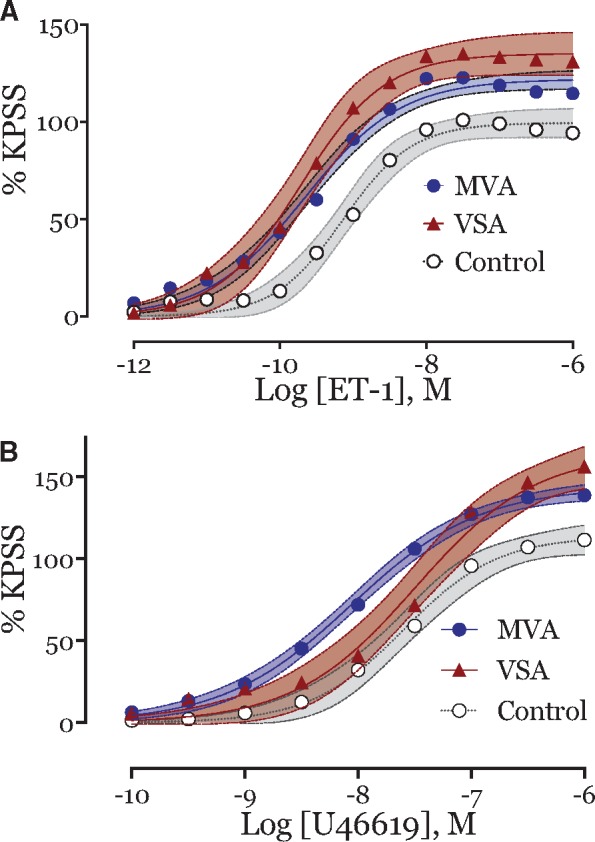
Cumulative concentration-response curves to constrictor agonists. (*A*) Increased maximum vasoconstriction to endothelin-1 in both microvascular angina subjects (blue circle, endothelin-1 *E*_max_ 121%, *n* = 54; *P* = 0.03) and vasospastic angina subjects (red triangle, endothelin-1 *E*_max_ 125%, *n* = 11; *P* = 0.02) vs. control subjects (○—white circle, endothelin-1 *E*_max_ 100%, *n* = 9; *P* = 0.02). No difference in endothelin-1 *E*_max_ between microvascular angina and vasospastic angina subjects (*P* = 0.397). Comparison of CCRC fit, *P* < 0.001. (*B*) Cumulative concentration-response curves to thromboxane analogue, U46619, in both microvascular angina subjects (blue circle, U46619 *E*_max_ 143%, *n* = 54; *P* = 0.01) and vasospastic angina subjects (red triangle, *E*_max_ 141%, *n* = 10; *P* = 0.04) vs. control subjects (○—white circle, endothelin-1 *E*_max_ 109%, *n* = 11). No difference in *E*_max_ between microvascular angina and vasospastic angina subjects (*P* = 0.932). Comparison of CCRC fit, *P* < 0.001. Each measure represents mean ± 95% confidence intervals for mean in shaded contours from CCRC best-fit.

All arteries responded in a concentration-dependent manner to both endothelin-1 (ET-1) and U46619. In all subjects, blood vessels were ≈50-fold more sensitive to the constrictor effects of ET-1 compared with the thromboxane agonist U46619 (median ET-1 *pIC*_50_ ET-1 9.6 vs. 7.9; 95% CI of median difference in *pIC*_50_ 1.4–1.9; Mann-Whitney U = 247, *P* < 0.001). Importantly, the maximum constrictor response to ET-1 was greater in MVA compared with the control group (121% vs. 100%; *P* = 0.03; *Figure 4A*). The maximum response to U46619 was also greater in MVA (143% vs. 109%; *P* = 0.01; *Table 3* and *Figure 4B*). The VSA group had similar patterns of increased vasoconstriction to both ET-1 (median 125% vs. 100%; *P* = 0.02) and U46619 (median 141 vs. 109%; *P* = 0.04). These were not significantly different from the MVA subjects (*Table 3* and *Figure 4*). The key findings of the study are summarized in the *Take home figure*.

**Take home figure:**
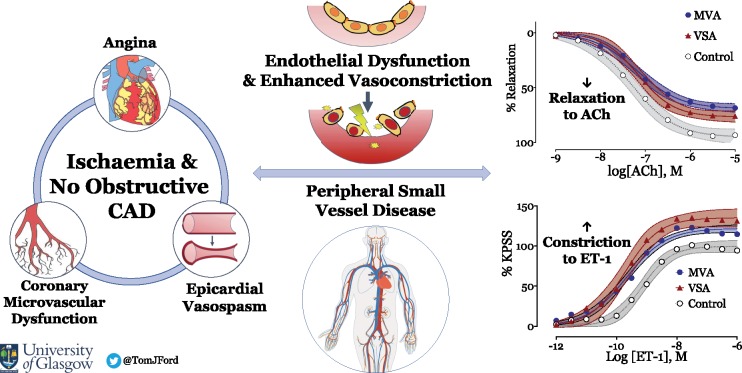
Systemic microvascular dysfunction in microvascular and vasospastic angina.

### Safety of biopsy procedure

No serious adverse events occurred following the gluteal biopsy. There were 5 (6.2%) cases of minor wound dehiscence post procedure without clinically significant wound infection. Wound healing occurred by secondary intention in all cases without additional treatment.

## Discussion

We have shown altered function of peripheral small arteries in patients with INOCA compared with control subjects. Specifically, both MVA and VSA patients had peripheral microvascular abnormalities characterized by reduced maximum relaxation to ACh (in keeping with endothelial dysfunction) and increased responses to vasoconstrictor stimuli. Vasoconstriction in response to ET-1 and the thromboxane agonist U46619 was greater in MVA and VSA patients. Overall, our results stimulate the provocative concept that patients with MVA or VSA are at risk of developing systemic small vessel disease. Our study helps explain the associations between coronary microvascular dysfunction and small vessel disease in other organs, such as the brain and kidney.^26^

To our knowledge, this is the first study to prospectively investigate patients with INOCA and objective invasive evidence of coronary microvascular function using wire myography to assess peripheral vascular function. These assessments were temporally approximated within a 4 week period. Furthermore, a control group of subjects with normal coronary microvascular function who underwent the same interventional diagnostic procedures were also included. This protocol included reference invasive coronary testing based on ESC^4^ and COVADIS^27^ diagnostic criteria for disorders of coronary artery function. We used validated questionnaires to classify angina symptoms as an eligibility criterion to avoid inclusion of patients with non-cardiac chest pain.^14^ All patients had non-obstructive coronary artery disease as determined by an FFR value >0.80. Patients were then diagnosed with either MVA or VSA based on the presence coronary microvascular dysfunction or significant epicardial constriction during provocation testing. The control group (comprising patients referred with chest pain proven not to have any abnormality in coronary artery function) was considered to be a more appropriate control population than volunteers without classification of coronary vascular function.

Wire myography is a reference technique for *in vitro* research of small resistance arteries and is well established in our laboratory. This method enables vascular mechanisms to be specifically investigated, which otherwise would not be possible *in vivo.* Our findings extend prior studies that identified abnormalities in peripheral vascular function in patients with both MVA^28^ and VSA.^29^ Microvascular angina is a disorder of coronary microvessels (≤400 μm)^30^ whereas VSA is a disorder of conduit arteries,^31^ and thus it is somewhat surprising that both groups have reduced relaxation to ACh compared with controls. Systemic endothelial dysfunction may simply be a secondary manifestation of coronary vascular dysfunction. Alternatively, it may be a primary feature reflecting underlying pathophysiology in both conditions. Our study was not powered to detect a difference between ACh *E*_max_ between MVA and VSA, so we cannot draw definitive conclusions about comparative endothelial function between the MVA and VSA groups. It would be expected that MVA patients would have reduced endothelial function compared with VSA patients although confirmation of this would require a much larger study. Notwithstanding the distinct differences in the pathogenesis of these disorders, endothelial dysfunction is implicated in the aetiology and progression of both these INOCA endotypes.^8^^,^^9^^,^^28^ Treatment with drugs that improve endothelial function and/or coronary blood flow have plausible symptomatic and physiological benefits to patients with either of these disorders.^32^

### Mechanisms underlying endothelial dysfunction and implications for clinicians

Our work provides new insights into the presence and mechanisms of systemic arteriolar dysfunction. The small arteries studied *in vitro* were from a different vascular bed from those implicated in the pathogenesis of coronary microvascular dysfunction. The cross-sectional study design limits understanding of the natural history and causality, and concomitant cardiovascular treatment is a potential confounder. As such, our findings are associative but the structural and functional changes that occur in human subcutaneous small arteries in response to vascular risk factors, such as diabetes mellitus and hypertension, have prognostic relevance^33^ and may be mirrored in other circulatory beds (e.g. heart and brain).^26^ Future studies should investigate microvascular structure/function relationships (e.g. media: lumen ratio using pressure myography) due to the known prognostic utility of this measurement.^34^

We did not measure nitric oxide (NO) and it is possible that bioavailable NO was not deficient, rather the mechanisms of vascular dysfunction occurred in the vascular smooth muscle cells (VSMCs). It is known that high doses of ACh provoke constriction via direct action on vascular smooth muscle muscarinic receptors in the coronary circulation. However, this effect does not occur in human small peripheral arterioles.^10^^,^^35^ Modulation studies in human small peripheral arterioles using exogenous NO donor (SNP) or a hyperpolarizing agent (pinacidil) including measurements of endothelial nitric oxide synthase (eNOS) support endothelial dysfunction as the primary cause of failure to relax to ACh.^36^ In our study, we observed appropriate *in vitro* vasorelaxation in response to the NO donor, SNP, consistent with normal responses to exogenous NO. The SNP-mediated effects are independent of NO derived from eNOS activation and accordingly have been considered as endothelial-independent vasorelaxation.^37^

Our results contrast with a study of forearm blood flow (FBF) that reported preserved ACh mediated relaxation in patients with Syndrome X (a historical term for angina with primary coronary microvascular dysfunction—one endotype of INOCA).^38^ Additionally, the forearm constrictor response to ET-1 was reduced in patients vs. controls possibly implicating ET_A_ receptor down-regulation in response to elevated ET-1 levels. Differences between *in vitro* and *in vivo* studies of human endothelial function are well recognized.^39^ Disparities between FBF and isolated resistance arteries may reflect physiological variations between vessels of differing circulatory origin; gluteal biopsies provide solely subcutaneous resistance arteries, whereas FBF occurs through larger muscular arteries flow with less significant resistance to flow from resistance arteries.

### Putative mechanisms of increased peripheral vasoconstriction

Our research findings stimulate the provocative concept that MVA and VSA associate with a generalized susceptibility to vasoconstrictor stimuli. We found enhanced contractile responses to both ET-1 and U46619 in small resistance arteries from patients. This finding may be due to endothelial dysfunction contributing towards an imbalance of bioavailable constrictor factors (ET-1, thromboxane A2, reactive oxygen species, and prostaglandin) which predominate over endothelium derived relaxing factors (nitric oxide (NO), prostacyclin ( PGI2) and endothelium derived relaxing factor (EDRF)). We recognize the potential for concomitant epicardial vasospasm in patients with coronary microvascular dysfunction and the potential for exaggerated microvascular constriction in patients with VSA.^40^ Removing the endothelium (denuding) in the arterioles of subjects from both groups would have allowed further insights into the mechanism of increased contractility.

Abnormalities vascular at VSMC level with pathological constrictor response to ACh in the smooth muscle is another alternative mechanism (also associated with oxidative stress). Vascular smooth muscle cells contain several sources of reactive oxygen species that mediate many pathophysiological processes such as growth, migration, apoptosis, and secretion of inflammatory cytokines.^41^ Indeed, hypercontraction of VSMCs is the proposed mechanism of epicardial VSA.^31^ Many patients with epicardial vasospasm are likely to have coronary microvascular dysfunction and exaggerated microvascular constriction as a cause of ischaemia. It is certainly plausible that generalized vascular smooth muscle responsiveness was enhanced and testing other constrictor agonists (e.g. noradrenaline) would have been informative. Our patients have coronary microvascular and/or epicardial propensity to vasospasm which can lead to ischaemia or even myocardial infarction.^42^ It is a plausible concept that vascular diseases including migraine, Raynaud’s and MVA are linked by a propensity to vasoconstriction with endothelial dysfunction.^43^^,^^44^

### Limitations

As with all case–control studies, the selection of a true ‘reference control’ group is a potential limitation. Within the constraints of any matching criteria, we approached consecutive patients with negative testing of coronary function with similar exposure to risk factors and confounders that were representative of the population at risk. Nevertheless, assignment of participants to the control group in this way may have introduced unmeasured confounding bias.

The diagnosis of MVA in our population followed established guidelines, however, the underlying pathophysiology is likely to differ between individual patients reflecting ‘real world’ practice. We recognise other even more in depth endotypes, such as the ′coronary slow flow phenomenon′ proposed by Tambe *et al.*^45^ and more recently by Beltrame's group.^46^ These patients characteristically have isolated increase in microvascular resistance at rest but preserved CFR. Another subgroup is ‘microvascular vasospasm’ proposed by Mohri *et al.*^47^ While respecting these distinct subgroups, our classification dichotomises patients with isolated epicardial vasospasm (VSA group) from patients with microvascular dysfunction ‘MVA group’, and importantly, these are defined to align with contemporary COVADIS criteria.^27^ We hope future studies extend our research unravelling the pathophysiology of distinct endotypes within cohorts with INOCA enabling a precision medicine approach.^30^

Despite the normalization process that serves to standardize each experiment, *in vitro* myography does not fully replicate physiological conditions *in vivo*. Considering the primary outcome of the maximum vasorelaxation response to ACh, we averaged the response of arterioles in order to increase accuracy and average within subject variation of microvascular function. Indeed, the same approach is established for other physiological measurements, including guidewire-based coronary thermodilution for measurement of coronary microvascular resistance.^16^^,^^17^ Additionally, vessel dissection, processing, and removal of perivascular fat may alter vascular function. Perivascular fat plays an important neurohormonal function which modifies vascular reactivity.^48^ Despite an experienced team of scientists, we could not extract viable vessels from all of the biopsies and a minority of samples provided only one or two vessels. This experience is typical in isometric tension recordings in human blood vessels. Therefore, a complete set of pharmacological experiments was not feasible in in all subjects (*Table 3*). We have described one of the largest series of myography experiments in a single study, and the largest to date in patients with INOCA. Nonetheless, the sample size is a potential limitation of our study inherent to the technically demanding procedure of gluteal fat biopsy and wire myograph protocol.

## Future directions

Recent trials have increased physician awareness of stable coronary syndromes beyond fixed luminal obstruction; half of patients may have ongoing angina despite technically successful percutaneous coronary intervention.^49^ The relationship between CAD, ischaemia, and angina is not straight forward and functional coronary disorders are relevant. Our work supports the call from a recent White Paper for more research into the diagnosis and management of INOCA.^1^

Endothelin-1 is a potent endogenous vasoconstrictor peptide. In this study, ET-1 was ≈50 times more potent than the thromboxane A_2_ analogue U46619. The increased constriction to ET-1 observed in the present study raises the question about whether interventions which reduce the effects of this peptide may, therefore, be useful in the treatment of such patients. An appreciable component of the coronary smooth muscle contractile response to ET-1 signalling mechanism is calcium antagonist-resistant.^50^ This suggests a role for selective ET receptor antagonists in the treatment of patients with INOCA. Further studies are required to assess whether selective ET-1 receptor modulation may be helpful in these patients.

In conclusion, we have identified peripheral endothelial dysfunction and enhanced vasoconstriction in MSA and VSA. These processes may involve ET-1. Small vessel damage in the heart may be part of a widespread phenomenon with INOCA patients at risk of other small vessel diseases. Overall, further research is required but this study reminds us to think of angina as a cardiac symptom of a systemic disease where treatment of the entire cardiovascular system (including basic lifestyle measures) should be encouraged.
